# Evaluation of thyroid function tests among children with neurological disorders

**DOI:** 10.3389/fendo.2024.1498788

**Published:** 2024-12-09

**Authors:** Haojie Meng, Francis Manyori Bigambo, Wei Gu, Xu Wang, Yang Li

**Affiliations:** ^1^ Department of Children Health Care, Children’s Hospital of Nanjing Medical University, Nanjing, Jiangsu, China; ^2^ Clinical Medical Research Center, Children’s Hospital of Nanjing Medical University, Nanjing, Jiangsu, China; ^3^ Department of Neurology, Children’s Hospital of Nanjing Medical University, Nanjing, Jiangsu, China

**Keywords:** neurological disorders, attention deficit disorder with hyperactivity, autism spectrum disorder, thyroid function tests, children

## Abstract

**Background:**

Thyroid hormones (THs) are essential for brain development. Numerous studies have identified significant links between thyroid dysfunction and cognitive function. However, research on the significance and necessity of thyroid function tests in diagnosis of neurological disorders is limited and subject to controversy.

**Methods:**

Our study employed a combination of meta-analysis and case-control design. For the meta-analysis, we conducted a systematic search of online databases for studies that compared thyroid function tests in children with neurological disorders to controls. In our case-control study, we recruited a total of 11836 children, comprising 7035 cases and 4801 healthy controls. Wilcoxon Rank Sum Test was used to determine characteristics of thyroid function between the cases and healthy controls. In order to exclude the false discovery rate (FDR), the Benjamini-Hochberg (BH) procedure is applied.

**Results:**

A total of 12 relevant literature sources were included in the meta-analysis. Compared with controls, free thyroxine (FT4) levels were significantly decreased in neurological disorders in meta-analysis (MD = -0.29, 95% CI: -0.50 to -0.09), whereas thyroid-stimulating hormone (TSH) levels showed no significant difference (MD = -0.07, 95% CI: -0.36 to 0.21). In our case-control study, levels of free thyroxine (FT4), total triiodothyronine (TT3), total thyroxine (TT4), and anti-thyroglobulin antibodies (TG-Ab) were notably reduced among individuals with neurological disorders, compared with healthy controls (*P*<0.001, *P<*0.001, *P*=0.036, *P*=0.006). However, thyroid-stimulating hormone (TSH) levels did not show any statistically significant differences among the cases and controls.

**Conclusions:**

Our research demonstrates that, in comparison to controls, children with neurological disorders exhibited a significant decrease in FT4 levels, while TSH levels remained unchanged. This finding provides a reference for potential serum marker of neurological disorders in children. Replication in future studies with the assessment of THs is needed to determine whether thyroid function should be included as a routine screening in these children.

## 1 Introduction

Thyroid hormones (THs) play a vital role in the development of human brain by regulating genes responsible for brain formation, growth, and structuring (1). THs are indispensable regulators within the human body, governing a multitude of metabolic and serving as pivotal determinants in the developmental processes of both the infant’s brain and body, affecting the functionality of most organ systems (2). Excessive secretion of THs can lead to agitation, irritability, and distractibility, while insufficient secretion may result in memory loss, as well as slowed speech and movement. It is currently thought by the psychiatric community that patients with hypothyroidism are more prone to exhibiting abnormal moods, reduced cognitive function and behavioural abnormalities (3). Thyroid dysfunction is closely associated with cognitive impairment and dementia (4), as well as Autism Spectrum Disorder (ASD), Attention Deficit Disorder with Hyperactivity (ADHD), depression (5), and schizophrenia (6).

Neurological disorders such as provisional and chronic tic disorder (TD) impact around 3% and 1% of the population, respectively, with greater susceptibility to children (7). Notably, males have a significantly higher incidence rate than females. TD often co-occurs with neurological conditions, with ADHD and obsessive-compulsive disorder being the most prevalent (8). ADHD was approximated to be 3.4% of the general population. There was an association between the exposure and an elevated risk of adverse outcomes in the future, including lower educational achievement, social challenges, substance abuse, and involvement in criminal activities (9). In developed countries, the prevalence of ASD was around 1.5% and it was strongly associated with various conditions, including mental, physical, functional challenges, and neurodevelopmental (10, 11).

THs are synthesized by the thyroid gland and transported through the bloodstream to various parts of the body. Research indicates that thyroid dysfunction during pregnancy may elevate the increasing risk of ADHD and ASD in children (12–15). However, several studies on the relationship between abnormalities of THs during pregnancy and neurological disorders in the offspring have not found the significant association (16, 17). Moreover, excessive thyroid hormone treatment during pregnancy has been linked to a higher likelihood of ADHD symptoms and behavioral challenges in offspring (18).

Research on the association between thyroid dysfunction and neurological disorders in children is limited, and consensus on this relationship has yet to be reached. These studies are also constrained by limitations of sample size. Our study is aimed to evaluate the characteristics of thyroid function tests between children with neurological disorders and healthy controls. Furthermore, we seek to explore whether thyroid function tests should be considered as recommended screening measures in clinical practice. Our research focuses on neurological disorders, including TD, ASD and ADHD.

## 2 Materials and methods

### 2.1 Meta search and inclusion criteria

We searched Embase, Cochrane Library, PubMed, and Web of Science for relevant literature. The literature review was conducted up to August 23, 2024, using the following search terms: [(Thyroid Function Tests OR Thyroid Hormones OR Thyrotropin) AND (Attention Deficit Disorder with Hyperactivity OR Autism Spectrum Disorder OR Tourette Syndrome) AND (Child, Preschool OR Children OR Adolescent)]. Inclusion criteria consisted of the following: A. Studies employed observational original researches, rather than case studies, reviews, books, letters, or conference papers. B. Studies provided subjects number in both neurological disorder cases and control groups, along with the mean and standard deviation (SD) or median and interquartile range for serum thyroid function test levels. C. Neurological disorder cases were limited to those with ADHD, ASD, or TD, excluding other mental disorders and organic diseases. D. Study subjects were children, not adults and experimental animals. E. Articles published after 2003. F. The full text of literature is available.

**Figure 1:**
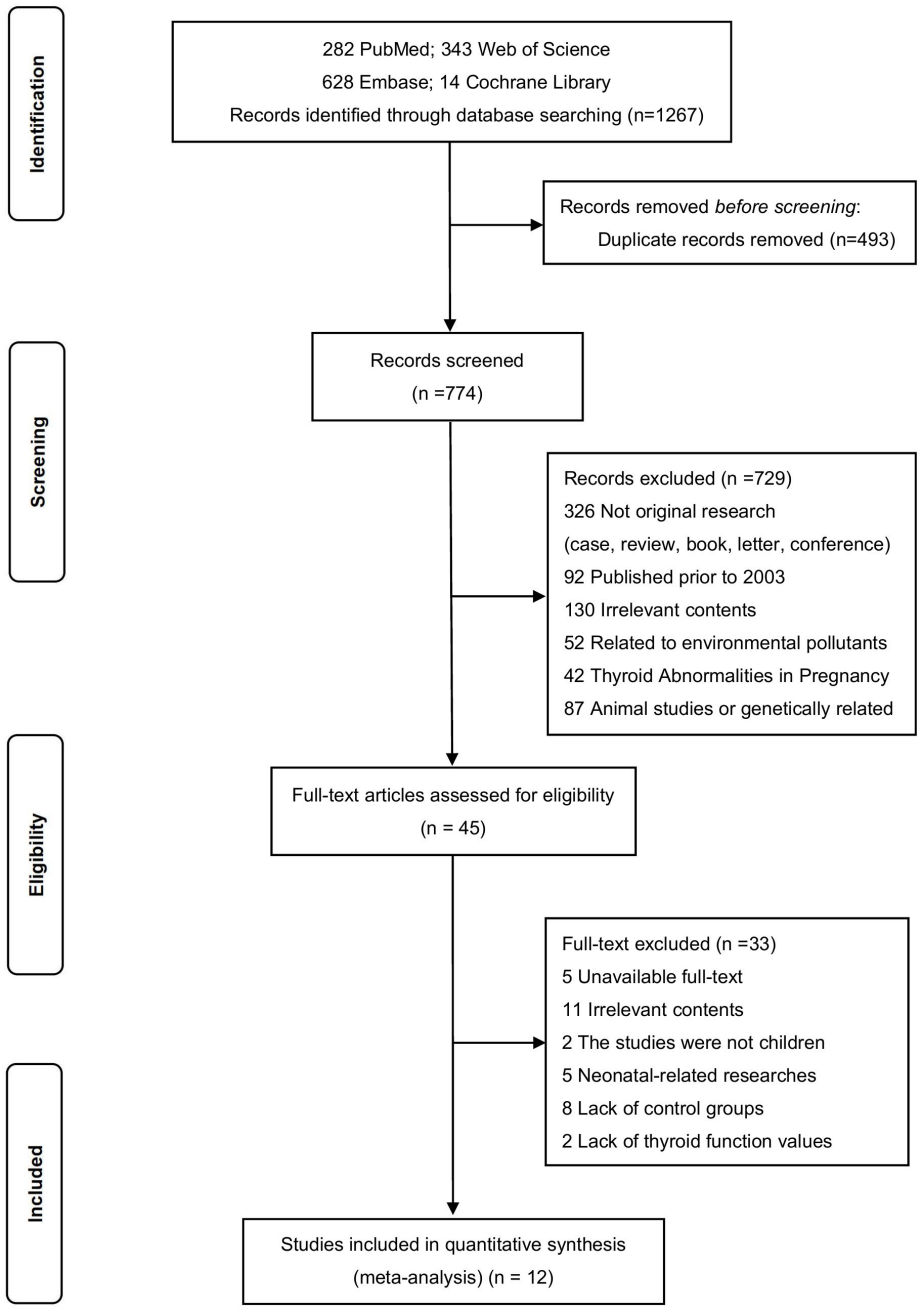
Meta-Analysis Diagram of Included Studies. **Figure 1** illustrates the process of the meta-analyses. We searched PubMed, Embase, Cochrane Library, and Web of Science for relevant literature. A total of 1,267 articles were retrieved, and after removing duplicates, 774 articles remained. After reviewing the titles and abstracts, 45 full-text articles were examined, and finally, 12 articles were included.

Two assessors participated in the literature screening and identification. In case of disagreement, selection was made through discussion with a third researcher (**Figure 1**). The Newcastle-Ottawa Scale (NOS) was used to evaluate the quality of the included studies, with scores ranging from 6 to 9 (19). The data extracted from the included studies encompassed the following: author name, published year, region, sample size, subjects age, gender, diagnostic criteria, measurement, method, and thyroid function test levels.

### 2.2 Clinical study design and participants

We performed a case-control study involving first-visit patients, from January 2017 to December 2021, at the Children’s Hospital of Nanjing Medical University. Our study included a total of 11836 children, comprising 7035 cases and 4801 healthy controls. The neurological disorders included were TD (n=1067), ADHD (n=4864), and ASD (n=1104). Our study was approved by the ethics committee of Children’s Hospital of Nanjing Medical University, in compliance with applicable laws and regulations. The guardians of the participating children obtained written informed consent.

### 2.3 Assessment of clinical thyroid function tests

Thyroid function tests were assessed at the Clinical Chemistry and Immunology Laboratory of the Children’s Hospital of Nanjing Medical University. Thyroid function tests, including free triiodothyronine (FT3), free thyroxine (FT4), thyroid-stimulating hormone (TSH), total triiodothyronine (TT3), total thyroxine (TT4), thyroid peroxidase antibody (A-TPO), and anti-thyroglobulin antibodies (TG-Ab), were quantified by Roche Elecsys automated electrochemiluminescence immunoassay. The thyroid function tests of all 11836 children in this study encompass measurements of FT3, FT4, and TSH, while data on TT3, TT4, A-TPO, and TG-Ab were available in 1435 children. At different ages of children, there are variations in the reference standards for thyroid function values. If the concentrations of A-TPO and TG-Ab level was below the limit of detection (LOD), the value was substituted with the LOD divided by the square root of two (20), and those exceeding the upper limit were substituted with the upper detection limit. Laboratory reference ranges for THs are shown in **Supplementary Table S1**.

### 2.4 Statistical analysis

The meta-analyses were conducted using the “meta” package in R (version 4.2.2) and STATA (version 18). For each study, the sample size, mean, and standard deviation (SD) of thyroid function tests were extracted for both the case and control groups. The effect sizes and the 95% confidence intervals (95% CI) were calculated, with mean difference (MD) employed as the measure of effect size. We used the *I²* statistic to evaluate the degree of heterogeneity. If values exceeded 50%, it indicated substantial heterogeneity. For the 9 studies pertaining to FT4, heterogeneity was minimal (*P* > 0.05, *I²* < 50%), and a fixed-effects model was selected. Conversely, for the 14 studies related to TSH, heterogeneity was considerable (*P* < 0.05, *I²* > 50%), necessitating the use of a random-effects model (21). To further examine the potential presence of publication bias, we conducted an Egger’s test.

SPSS (version 26.0) and R (version 4.2.2) were applied to analyze the data, in our clinical case-control study. Wilcoxon Rank Sum Test was used to determine characteristics of THs between the cases and healthy controls. A value of *P*<0.05 was accepted as statistically significant. In order to reduce the number of false positives and enhance the credibility of the results, we applied multiple testing corrections using the False Discovery Rate, the Benjamini-Hochberg (BH) method. In addition, to address the confounding effects of age and gender, we performed the Wilcoxon Rank Sum Test by age and gender-specific analysis. Descriptive statistics were presented as median (IQR) or mean ± standard deviation (SD), along with the number (n) and frequency (%) depending on the normality of the data. After employing a random forest model to adjust for potential confounding factors such as age and gender, we performed a receiver operating characteristic (ROC) analysis to validate the differences in FT4 levels between children with neurological disorders and the control group. The random forest model and ROC analysis were implemented using the “randomForest” and “pROC” packages in R (version 4.2.2).

## 3 Results

### 3.1 Meta analysis

**Figure 2:**
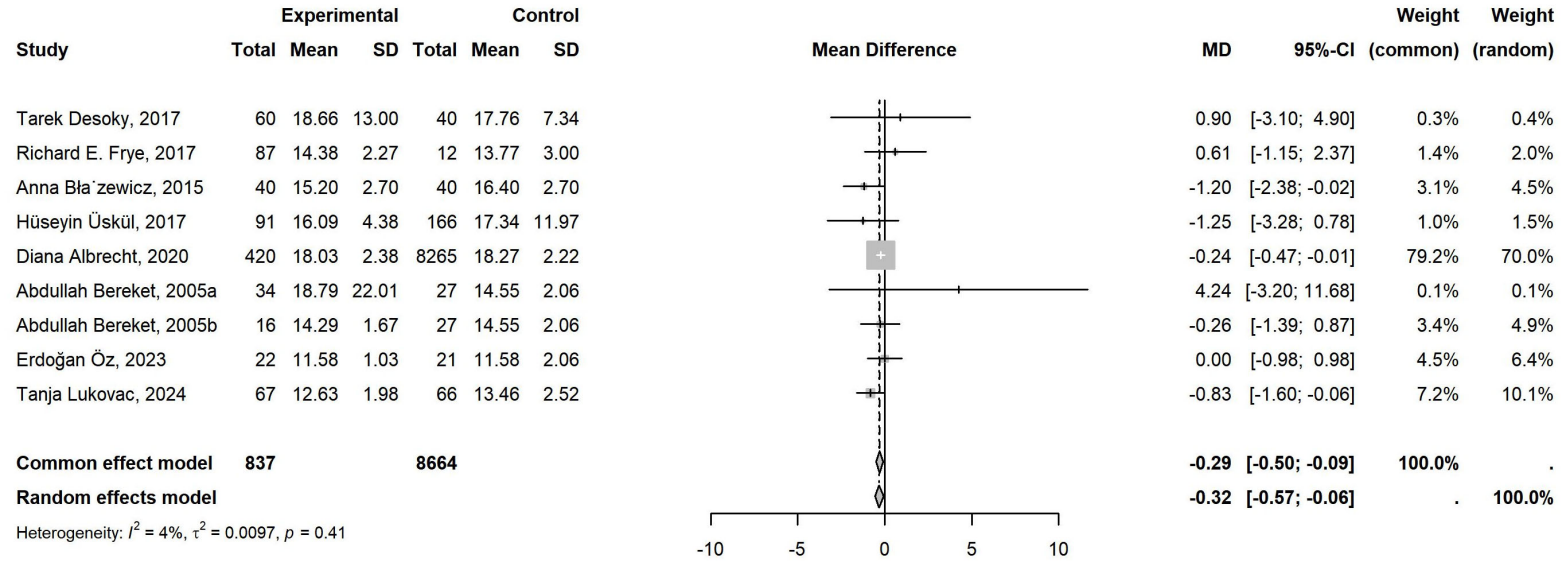
Meta-Analysis of FT4 levels in Children with Neurological Disorders compared to Healthy Controls. A total of 9 studies of FT4 comprising 837 cases and 8664 controls were included in the final meta-analysis. The heterogeneity was low (*P*=0.41>0.05, *I²*=4%<50%), so a common-effects model was used. Compared to the controls, the FT4 levels in the cases were significantly lower, with statistical significance (MD = -0.29, 95% CI: -0.50 to -0.09).

**Figure 3:**
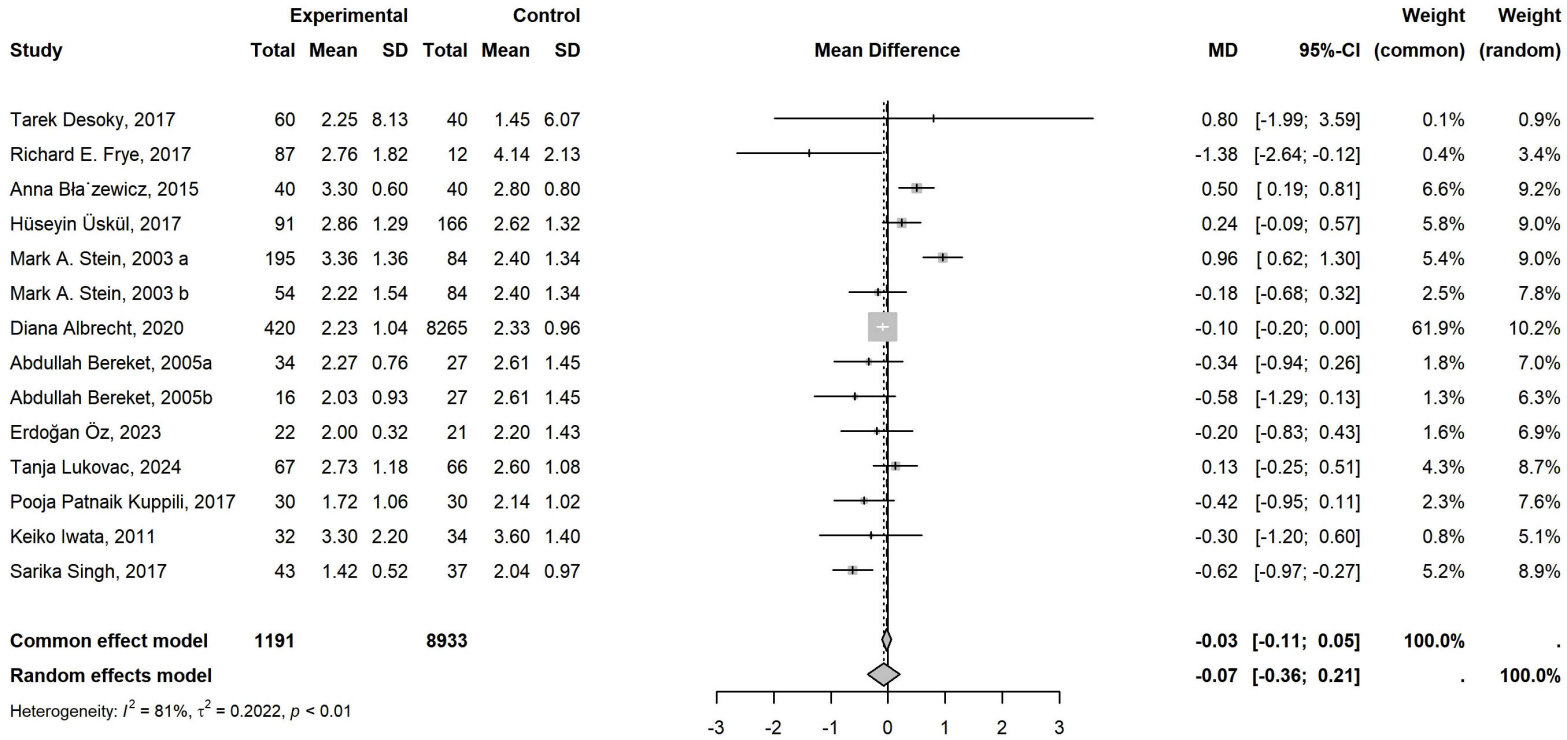
Meta-Analysis Comparing TSH Levels in Children with Neurological Disorders and Healthy Controls. 14 studies of TSH, involving 1191 cases and 8933 controls, were included in the final meta-analysis. Given the low heterogeneity (*P*<0.01, *I²*=81%>50%), a random-effects model was applied. When compared to the controls, there was no statistically significant difference in TSH levels in the cases (MD = -0.07, 95% CI: -0.36 to 0.21).

12 literature was included in the meta-analysis. Of these, 9 focused on FT4, comprising 837 cases, while 14 investigated TSH, involving 1,191 cases. **Table 1** showed the detailed characteristics of the included literature. The 9 studies related to FT4 exhibited low heterogeneity (*P*=0.41, *I²*=4%), and the common-effects model was employed to analyze the overall effect. A notable decline in FT4 levels was evident in children with neurological disorders when compared to the control group. (MD = -0.29, 95% CI: -0.50 to -0.09) (**Figure 2**). However, the 14 studies related to TSH exhibited high heterogeneity (*P*<0.01, *I²*=81%), which necessitated the use of the random-effects model. No statistically significant difference was identified in TSH levels between individuals with neurological disorders and healthy controls (MD = -0.07, 95% CI: -0.36 to 0.21) (**Figure 3**).

**Table 1:** Characteristics of the literature included in the meta-analysis.

Author	Year	Region	Sample size	Age(years)	Gender	Diagnostic Criteria	Measurement method	Thyroid Function Mean ± SD		Results	NOS score
			Patients/ Controls	Mean ± SD	No.males(%)			FT4 pmol/L	TSH uIU/mL		
Tarek Desoky (15)	2017	Egypt	60/40	7.03 ± 2.34 7.91 ± 3.21	55 (92.6%) 20 (50.0%)	CARS	ELISA	18.66 ± 13.00 17.76 ± 7.34	2.25 ± 8.13 1.45 ± 6.07	The overall measurement results show significant higher mean serum TSH levels among autistic children when compared with the control group (p,0.05 for all).	8
Richard E. Frye (22)	2017	Arkansas, America	87/12	6.83 ± 3.08 8.17 ± 5.17	70(80.5%) -	ADOS ADI-R	ELISA	14.38 ± 2.27 13.77 ± 3.00	2.76 ± 1.82 4.14 ± 2.13	TSH did not differ from controls; FT4 slight higher than control F(1,94)=5.19, p=0.03.	6
Anna Bła ˙zewicz (23)	2015	Lublin, Poland	40/40	7.2 9.9	40(100%) 40(100%)	CARS	accredited diagnostic labs (Lublin, Poland)	15.2 ± 2.7 16.4 ± 2.7	3.3 ± 0.6 2.8 ± 0.8	Statistically significant lower levels of fT3 and fT4 and higher levels of TSH were found in the autistic group when compared with the control group.	8
Hüseyin Üskül	2017	Istanbul	91/116	9.0 ± 2.5 9.7 ± 3.1	68(74.7%) 79(68.1%)	DSM-IV	–	16.09 ± 4.38 17.34 ± 11.97	2.86 ± 1.29 2.62 ± 1.32	No statistically significant difference was found between the groups.	6
Mark A. Stein (24)	2003	America	195/84 (ADHD–CT)	8.9 ± 3.1 8.7 ± 3.04		DSM-IV	radioimmunoassay (Diagnostic products, Los Angeles, CA)		3.36 ± 1.36 2.40 ± 1.34	Thyroxine concentrations within the normal range were differentially associated with ADHD–Combined Type compared to ADHD–Predominantly Inattentive	6
			54/84 (ADHD–PI)	10.6 ± 3.3 8.7 ± 3.04		DSM-IV			2.22 ± 1.54 2.40 ± 1.34		
Diana Albrecht (25)	2020	German	420/8265	10.63 ± 2.75 8.8 ± 4.00	336 (80.0%) 4129 (49.96%)	ICD-10	electrochemiluminescence (Elecsys 2010, Roche Diagnostics, Mannheim, Germany)	18.03 ± 2.38 18.27 ± 2.22	2.23 ± 1.04 2.33 ± 0.96	We found a significant positive association between fT3 and continuously assessed ADHD symptoms in children.	9
Abdullah Bereket (26)	2005	Istanbul	34/27 (ADHD)	7.68 ± 3.20 9.80 ± 4.01	23 (67.6%) 13(48.1%)	DSM-5 DBDRS	the Architect CI-16200 (Abbott Diagnostics, Abbott Park, IL, USA)	18.79 ± 22.01 14.55 ± 2.06	2.27 ± 0.76 2.61 ± 1.45	Thyroid hormones and ntibodies were also examined among the three groups. We found no significant difference among the groups.	8
			16/27 (ASD)	7.88 ± 5.18 9.80 ± 4.01	10(62.5%) 13(48.1%)	CARS	the Architect CI-16200 (Abbott Diagnostics, Abbott Park, IL, USA)	14.29 ± 1.67 14.55 ± 2.06	2.03 ± 0.93 2.61 ± 1.45		
Erdoğan Öz (27)	2023	Turkey	22/21	8.3 5.0	14 (63.6%) 11 (52.4%)	DSM-V		11.58 ± 1.03 11.58 ± 2.06	2.0 ± 0.32 2.2 ± 1.43	No statistically significant difference was found between the groups.	6
Tanja Lukovac (28)	2024	Serbia	67/66	—	67 (100%) 66 (100%)	DSM-V	Immunoassay System Access, Beckan Coulter, Brea, California, USA	12.63 ± 1.98 13.46 ± 2.52	2.73 ± 1.18 2.60 ± 1.08	The likelihood of suffering from ADHD was lower when FT4 levels were elevated. The serum TSH level was significantly and independently associated with the diagnosis of ADHD.	6
Pooja Patnaik Kuppili (29)	2017	New Delhi	30/30	9.47 ± 2.43 10.30 ± 2.79	28(93.3%) 28(93.3%)	CPRS-R:S	Chemiluminescent Immunometric Assay		1.72 ± 1.06 2.14 ± 1.02	Serum total Thyroxine was significantly lower in cases of ADHD compared to controls, while no difference was found in TSH	7
Keiko Iwata (30)	2011	Japan	32/34	12.3 ± 3.2 12.4 ± 2.6	32 (100%) 34 (100%)	DSM-IV SCID	ELISA kit (R&D Systems, Inc., Minneapolis, MN, USA)		3.3 ± 2.2 3.6 ± 1.4	There were no significant differences in TSH levels between autistic and control	9
Sarika Singh (31)	2017	America	43/37	—	43 (100%) 37 (100%)	ADOS ADI-R	Myriad Rules-Based Medicine (RBM; Austin TX)		1.42 ± 0.52 2.04 ± 0.97	TSH levels were significantly lower in ASD boys. TSH levels were negatively correlated with the ADOS subdomain scores.	6

FT3, free triiodothyronine, (pmol/L); FT4, free thyroxine, (pmol/L); TSH, thyroid-stimulating hormone, (uIU/mL); ADHD, Attention Deficit Disorder with Hyperactivity; ASD, Autism Spectrum Disorder; CARS, Childhood Autism Rating Scale; DSM-IV, The Diagnostic and Statistical Manual of Mental Disorders IV; ICD-10, The International Statistical Classification of Diseases and Related Health Problems 10th Revision; ADOS, Autism Diagnostic Observation Schedule; ADI-R, Autism Diagnostic Interview-Revised; DBDRS, Disruptive Behavior Disorders Rating Scale; CPRS-R:S, Conners’ Parent Rating Scale –Revised short; SCID, Structured Clinical Interview for DSM-5 Disorders; ELISA, enzyme-linked immunosorbent assay; NOS, Newcastle Ottawa Scale.

**Figure 4:**
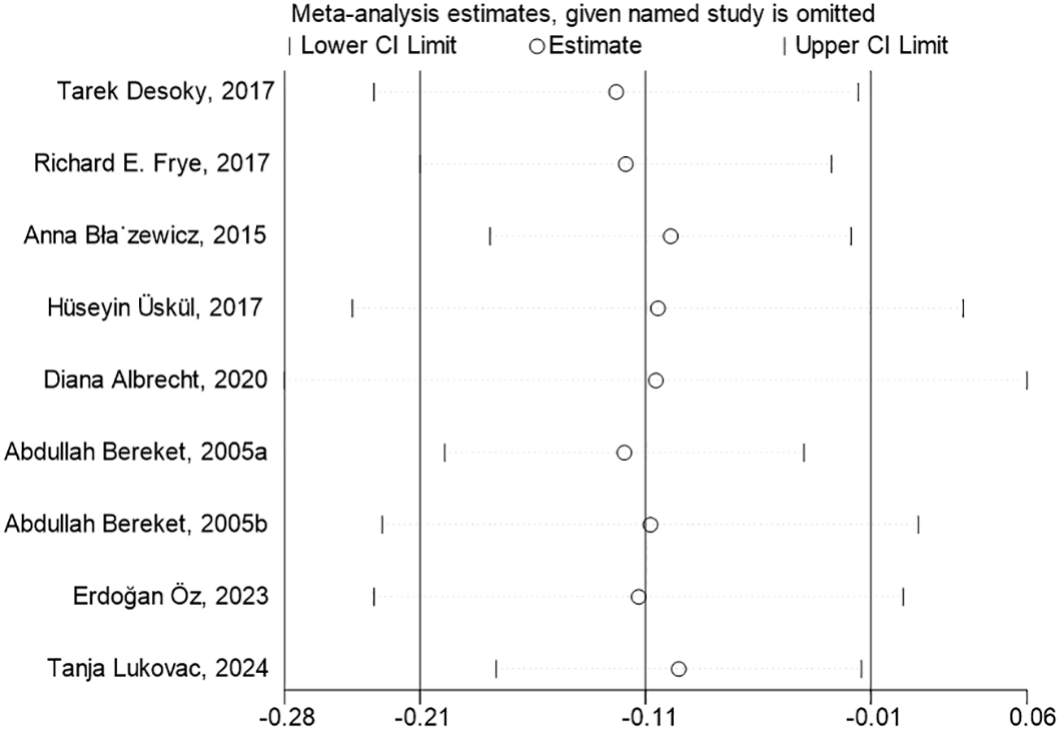
Sensitivity Analysis of FT4 Levels in Children with Neurological Disorders Compared to Healthy Controls. In the sensitivity analysis of the meta-analysis, the estimates from the 9 studies on FT4 levels remained within the 95% confidence intervals throughout all iterations.

**Figure 5:**
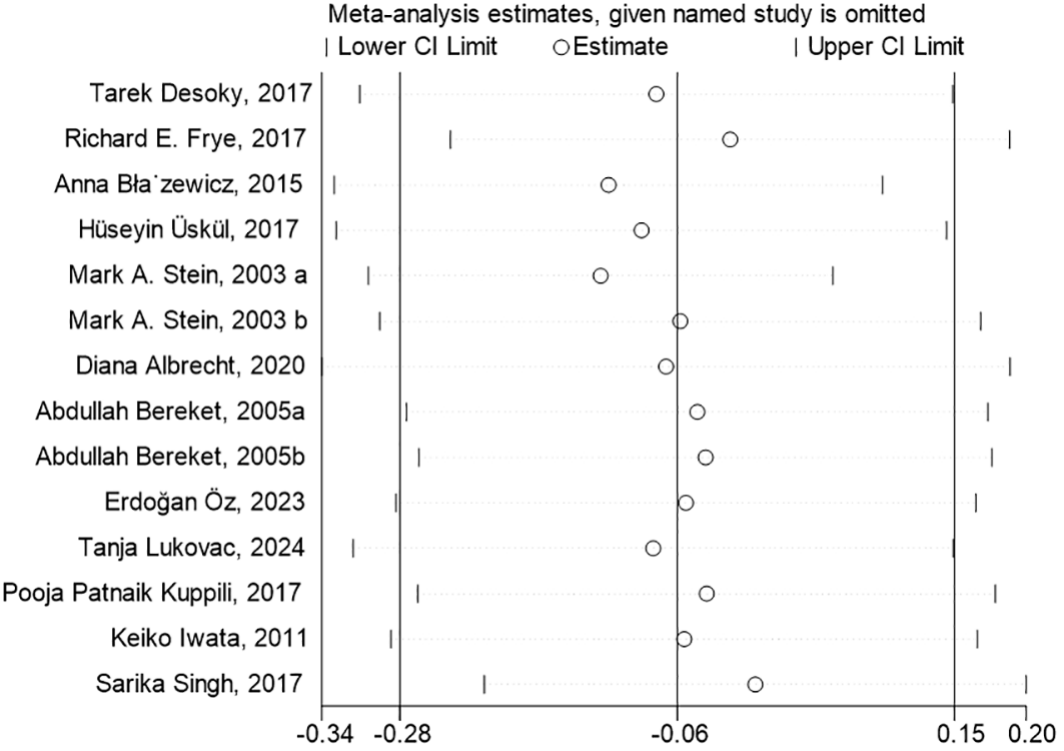
Sensitivity Analysis of TSH Levels in Children with Neurological Disorders Compared to Healthy Controls. The estimates from the 14 studies, in the sensitivity analysis of the meta-analysis, on TSH levels remained within the 95% confidence intervals throughout all iterations.

To conduct the sensitivity analysis, we employed a one-by-one elimination method with a random-effects model for both the FT4 and TSH analyses. The results demonstrated that the estimates remained within the 95% confidence intervals across all iterations, indicating that the overall effect sizes were robust and stable (**Figures 4**, **5**). The results of the Egger’s tests did not indicate the presence of publication bias in the FT4 (*P*=0.708) and TSH (*P*=0.834) analyses.

### 3.2 Clinical study

#### 3.2.1 Basic characteristics

In our case-control study, 11836 subjects were recruited in our study, comprising 7035 cases and 4801 healthy controls. **Table 2** presents a comparison of the demographic characteristics of children with neurological disorders and healthy controls. In our study, we primarily focused on three common neurological disorders: ADHD, ASD, and TD, with ADHD accounting for 69.14%, ASD for 15.69%, and TD for 15.17%. Demographic characteristics of the aforementioned children are summarized in **Supplementary Table S2**.

**Table 2:** Demographic characteristics and thyroid function levels of children with neurological disorders compared with healthy controls.

	Cases (Mean ± SD)	Healthy (Mean ± SD)	*P*
	n=7035	n=4801	
Age (years)	6.53 ± 2.72	6.34 ± 3.62	**0.003**
Gender			**<0.001**
Male	5680 (80.74%)	2771 (57.72%)	
Female	1355 (19.26%)	2030 (42.28%)	
Thyroid function levels			
FT3 (pmol/L)	6.78 ± 1.26	6.70 ± 1.15	**0.001**
FT4 (pmol/L)	18.10 ± 3.08	18.61 ± 2.73	**<0.001**
TSH (uIU/mL)	2.88 ± 1.64	2.88 ± 1.87	0.975
TT3 (nmol/L)	2.48 ± 0.55	2.61 ± 0.53	**<0.001**
TT4 (nmol/L)	110.52 ± 21.02	113.03 ± 23.16	**0.036**
A-TPO (IU/mL)	12.85 ± 29.29	17.09 ± 49.36	0.063
TG-Ab (IU/mL)	18.84 ± 81.63	51.33 ± 278.95	**0.006**

*P <*0.05 was considered statistically significant. FT3, free triiodothyronine, (pmol/L); FT4, free thyroxine, (pmol/L); TSH, thyroid-stimulating hormone, (uIU/mL); TT3, total-triiodothyronine, (nmol/L); TT4, total-thyroxine, (nmol/L); A-TPO, thyroid peroxidase antibody, (IU/mL); TG-Ab, anti-thyroglobulin antibodies, (IU/mL).Bolded P-values indicate statistical significance.

#### 3.2.2 Thyroid function tests

**Table 2** presents a comparison of the characteristics of children with neurological disorders and healthy controls. A notable reduction in the levels of FT3, FT4, TT3, TT4 and TG-Ab was observed when comparing the subjects with healthy controls. (*P=*0.001, *P<*0.001, *P*<0.001, *P*=0.036, *P*=0.006), while TSH and A-TPO levels did not show any statistically significant differences (*P*=0.975, *P*=0.063).

**Table 3** presents a comparative analysis of thyroid function test characteristics in children with specific neurological disorders and healthy cases. Following the False Discovery Rate multiple corrections of the *p*-Values, the levels of FT4 exhibited a significant reduction in children with ADHD (*P*<0.001), TD (*P*<0.001), and ASD (*P*<0.001) compared with the healthy controls. Additionally, TT3 and TT4 levels were significantly reduced in children with TD (*P*<0.001, *P*=0.020) and ADHD (*P*<0.001, *P*=0.012), while no significant difference was observed in ASD (*P*=0.453*, P*=0.214). Furthermore, in the False Discovery Rate correction, TG-Ab levels were significantly decreased in children with TD (*P*<0.001) and ASD (*P*<0.001) compared with the healthy controls. However, no statistically significant differences in FT3 levels were observed between children with TD and those with ADHD (*P*=0.065 and *P*=0.069, respectively).

**Table 3:** Laboratory characteristics of children including specific neurological disorders cases and healthy cases.

	Median (IQR)		*P* ^a^	*P* ^b^
	Healthy cases (n=4801)	TD cases (n=1067)		
FT3 (pmol/L)	6.670 (6.120-7.240)	6.720 (6.190-7.310)	**0.042**	0.065
FT4 (pmol/L)	18.470 (16.860-20.100)	17.880 (16.340-19.460)	**<0.001**	**<0.001**
TSH (uIU/mL)	2.570 (1.860-3.510)	2.640 (1.940-3.540)	0.128	0.173
TT3 (nmol/L)	2.590 (2.298-2.940)	2.400 (2.080-2.635)	**<0.001**	**<0.001**
TT4 (nmol/L)	113.200 (98.363-125.800)	108.650 (93.880-118.925)	**0.012**	**0.020**
A-TPO (IU/mL)	9.670 (7.360-12.710)	10.710 (8.110-13.540)	0.055	0.080
TG-Ab (IU/mL)	13.000 (10.358-15.103)	11.820 (7.071-13.720)	**<0.001**	**<0.001**
	Healthy cases (n=4801)	ADHD cases (n=4864)		
FT3 (pmol/L)	6.670 (6.120-7.240)	6.690 (6.150-7.270)	**0.046**	0.069
FT4 (pmol/L)	18.470 (16.860-20.100)	17.910 (16.500-19.470)	**<0.001**	**<0.001**
TSH (uIU/mL)	2.570 (1.860-3.510)	2.640 (1.910-3.580)	**0.010**	**0.018**
TT3 (nmol/L)	2.590 (2.298-2.940)	2.400 (2.173-2.680)	**<0.001**	**<0.001**
TT4 (nmol/L)	113.200 (98.363-125.800)	109.650 (96.430-121.075)	**0.006**	**0.012**
A-TPO (IU/mL)	9.670 (7.360-12.710)	9.970 (6.845-12.630)	0.597	0.627
TG-Ab (IU/mL)	13.000 (10.358-15.103)	12.440 (10.435-15.385)	0.333	0.400
	Healthy cases (n=4801)	ASD cases (n=1104)		
FT3 (pmol/L)	6.670 (6.120-7.240)	6.840 (6.253-7.380)	**<0.001**	**<0.001**
FT4 (pmol/L)	18.470 (16.860-20.100)	17.815 (16.513-19.248)	**<0.001**	**<0.001**
TSH (uIU/mL)	2.570 (1.860-3.510)	2.435 (1.750-3.410)	**0.004**	**0.008**
TT3 (nmol/L)	2.590 (2.298-2.940)	2.570 (2.305-2.835)	0.410	0.453
TT4 (nmol/L)	113.200 (98.363-125.800)	110.200 (98.075-121.750)	0.163	0.214
A-TPO (IU/mL)	9.670 (7.360-12.710)	9.860 (6.385-12.580)	0.322	0.398
TG-Ab (IU/mL)	13.000 (10.358-15.103)	7.071 (7.071-13.015)	**<0.001**	**<0.001**

TD, tic disorder, ADHD, Attention Deficit Disorder with Hyperactivity; ASD, Autism Spectrum Disorder;

*P <*0.05 was considered statistically significant. ^a^Wilcoxon Rank Sum Test. ^b^Benjamini-Hochberg test.

Concentrations of A-TPO and TG-Ab below the lower detection limit were replaced with values equal to the detection limit divided by the square root of two. Concentrations of A-TPO and TG-Ab above the upper detection limit were replaced with the upper detection limit.Bolded P-values indicate statistical significance.

Taking gender into account, we compared neurological disorders with healthy individuals by gender (**Supplementary Table S3**). After False Discovery Rate correction, statistically significant differences were observed in the level of FT4 in all neurological disorders in both female and male patients compared with healthy controls. Moreover, significant variations in TT3 levels were evident in both male and female patients with TD and ADHD, except ASD in male patients, which revealed no significant difference compared with the controls. Furthermore, statistically significant differences in TG-Ab levels were noted in male and female patients with ASD compared with healthy children.

Considering age as a factor, we conducted comparisons between individuals with neurological disorders and their healthy counterparts by age (**Supplementary Table S4**). We categorized age into five groups based on the stage of development: infancy (0-1 year), toddlerhood (1-3 years), preschool age (3-7 years), school age (7-11 years), and adolescence (11-18 years) groups. After False Discovery Rate correction, in the infancy group, no statistically significant differences were identified between the groups with and without neurological disorders, when compared with the healthy controls. In the toddlerhood group, TG-Ab levels were significantly reduced in ASD patients (7.071, IQR=7.071-13.000 IU/mL) in comparison with the healthy controls. Furthermore, when comparing with healthy controls, in the preschool, school-age, and adolescent groups, statistically significant differences in FT4, TT3, and TG-Ab levels were observed across all neurological disorders, except for TT3 level in ASD patients.

#### 3.2.3 ROC analysis in FT4

**Figure 6:**
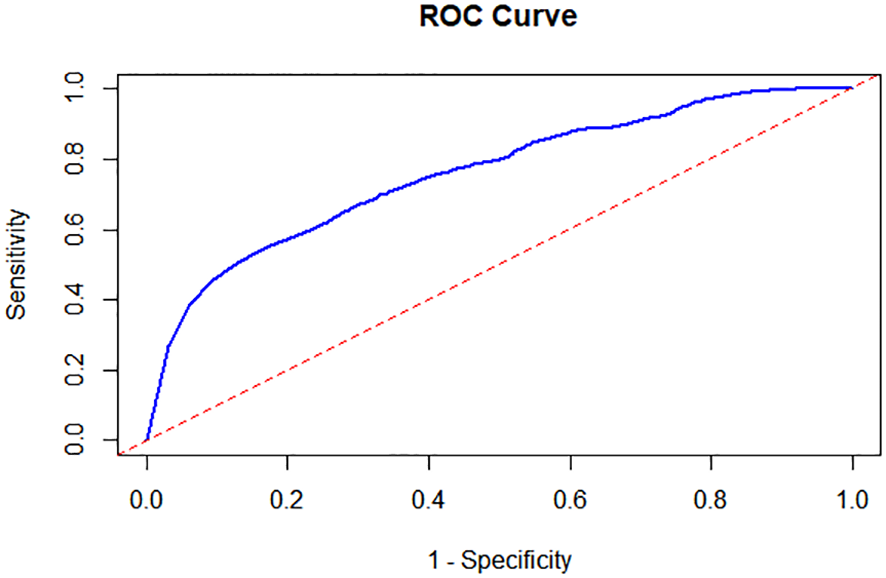
Receiver Operating Characteristic (ROC) Analysis in FT4 between the Cases and the Controls. Following the implementation of a random forest model, the Area Under the Curve (AUC) was 0.76 and the 95% confidence interval for the AUC ranged from 0.75 to 0.77.

Following the implementation of a random forest model to account for confounding variables, including age and gender, we observed that the Area Under the Curve (AUC) was 0.76 (**Figure 6**). This value signifies a robust classification capability of the model. Furthermore, the 95% confidence interval for the AUC ranged from 0.75 to 0.77, highlighting the model’s strong ability to distinguish between the FT4 levels of children with neurological disorders and those in the control group. These results not only affirm the model’s effectiveness but also suggest its potential utility in clinical settings for early identification and differentiation of children’s neurological conditions based on FT4 levels.

## 4 Discussion

Our research in pediatric neurological disorders focuses primarily on three common conditions: ADHD, ASD and TD. These three conditions are among the most prevalent and illustrative neurodevelopmental disorders in pediatrics. With the development of society, the prevalence of these disorders has increased, leading to increased awareness among parents regarding their children’s neuropsychological and behavioral health. The etiology of these disorders remains unclear and involves a complex interplay of abnormal brain structure, genetic predisposition, environmental influences, and social factors (9, 10). Diagnosis of these conditions is currently based almost exclusively on questionnaires and structured interviews, according to the criteria set by the International Classification of Diseases, 11th edition (ICD-11) and the Diagnostic and Statistical Manual of Mental Disorders, 5th edition (DSM-5) (32) being the widely accepted standards. However, these diagnostic criteria rely heavily on clinician expertise, which introduces a degree of subjectivity. Objective laboratory tests, such as serological or imaging assessments, are particularly lacking in the diagnosis of such neurological disorders. In our study, we aim to explore the characteristics of thyroid function tests in children with neurological disorders compared to healthy controls, with the goal of investigating whether thyroid function assessment should be recommended as a standard screening tool in the clinical evaluation of these disorders.

THs are responsible for overseeing essential metabolic processes that support normal growth and development (33) and developing the fetal brain (34). THs boost the performance of amino-acyl-tRNA synthetases, enhancing their activity across a wide range of amino acids within various bodily organs and tissues (3). The decline in neuronal networks, crucial for brain maturation and programmed cell death in the brain, is linked to thyroid-dependent apoptosis (35). THs affect various facets of neural development, including the proliferation and differentiation of neuronal precursors, myelination and neuronal migration, and so on (36). THs have potential regulatory effects on motor, cognitive, and emotional functions (37, 38). Thyroid dysfunction is associated with kidney diseases (39), cardiovascular diseases (40), diabetes, dementia (4), and others by renal, systemic hemodynamic, metabolic, and cardiovascular effects (39). Total thyroxine (TT4) and triiodothyronine (TT3) are particularly vital for the central nervous system, as thyroid hormones contribute to brain maturation during all stages of gestation (41).

Numerous studies have demonstrated that insufficient thyroid hormone levels during intrauterine development can impact the neurodevelopment of offspring and also increase their susceptibility to ADHD (12, 42–44). Currently, a considerable number of clinical studies are concentrating on the relationship between thyroid hormone levels during pregnancy and the occurrence of neurological disorders in offspring. Nevertheless, no consensus has been reached. A meta-analysis revealed a correlation between maternal hyperthyroidism and an elevated risk of ADHD and epilepsy in offspring (45). Furthermore, maternal hypothyroidism has been associated with an elevated risk of ADHD and ASD in children. A Danish study demonstrated that hyperthyroidism during pregnancy rises the hazard of ADHD in offspring (44). However, some studies failed to find out a correlation between thyroid dysfunction during pregnancy and neurodevelopmental outcomes (16, 46). The study involving children born in Orange County (47) and the study in Southern California (48) did not find an association between TSH levels and regressive ASD. Interestingly, there are reports suggesting that children of mothers who were overtreated for thyroid abnormalities exhibited more ADHD symptoms and behavioral difficulties (18).

Thyroid dysfunction is often comorbid with neurological disorders in children (13). A study conducted by R.E. Weiss revealed that 48% to 73% of children diagnosed with resistance to thyroid hormone syndrome also exhibited symptoms of ADHD (49). Research from Taiwan revealed that some children were diagnosed with ADHD before any detectable thyroid dysfunction (13).

However, there is a paucity of research into the relationship between serum THs levels in children and neurological disorders. The results of a case-control study indicated that serum total thyroxine (T4) in children with ADHD were markedly lower than those observed in healthy controls (29). Similarly, studies conducted by Tarek Desoky (50) and Anna Błażewicz (23) revealed that children with ASD exhibited significantly elevated mean serum TSH levels in comparison to the control group. Despite TSH levels in ASD children being within the normal range, elevated TSH concentrations have been linked to lower cognitive function in some studies (50, 51). Research conducted in Serbia showed that higher serum FT4 levels were associated with a reduced likelihood of having ADHD (28). Yet many studies have not identified a correlation between TH levels and neurological disorders (17, 26, 27, 29). Additionally, neurological disorders may also be related to abnormalities in the hypothalamic-pituitary-adrenal axis (52). A study involving 107 children (42 females and 65 males) suggests distinct associations between TD and maternal autoimmune conditions, particularly hypothyroidism, indicating the potential presence of autoimmunity in these children (53).

The results of our meta-analysis indicate a significant correlation between decreased FT4 levels and neurological disorders. However, the analysis revealed no statistically significant correlation between TSH levels and the prevalence of neurological disorders. The results of our clinical study indicated statistically significant reductions in FT4, TT3, and TT4 levels in patients with TD, ADHD, and ASD. Additionally, reduced TG-Ab levels were observed in children with TD and ASD. Interestingly, ADHD patients displayed elevated TSH levels, while individuals with ASD had higher FT3 levels. These findings suggest that thyroid dysfunction may influence neurological function in children.

To minimize the impact of potential confounding factors, such as gender and age, we separately compared the characteristics of thyroid function tests among children with neurological disorders and healthy controls by age and gender. In the gender-specific analysis, it’s noteworthy that we did not observe significantly distinct patterns. The age-specific analysis revealed no statistically significant differences in the infant group. The absence of significant differences in infancy suggests that thyroid function may not play a prominent role in the etiology of these disorders during this early developmental stage, although such result could also be attributed to the small sample size for infants. Furthermore, significant differences in TT3, FT4, and TG-Ab levels were caught in pre-school, school-age, and adolescence groups implying that the influence of thyroid function increases as individuals grow and develop. A German study (25) also suggested that the relationship between thyroid function biomarkers and ADHD risk may be influenced by physical maturity.

Combining our meta-analysis and clinical study, we reached a unified conclusion: FT4 levels showed a significant decrease in children with neurological disorders, whereas no statistically significant difference was observed in TSH levels when compared to healthy controls. A meta-analysis, including three prospective birth cohorts from Spain, the Netherlands and the UK, found that FT4 is a reliable indicator of foetal thyroid status in early pregnancy. A low FT4 was found to be significantly associated with a lower IQ and an increased prevalence of ASD in the offspring (49). Research evidence has shown that the incidence of autism spectrum disorder (ASD) is higher in infants with low T4 levels at birth (47). Further studies are required to replicate and fully understand the scope of these findings.

Although thyroid function tests are widely used in clinical practice for diagnosing conditions such as hyperthyroidism, hypothyroidism, and thyroid cancer, they have several limitations. Firstly, despite the prevalent use of enzyme-linked antibodies in enzyme-linked immunosorbent assays (ELISA) across most laboratories, variations in equipment and skill levels can lead to inconsistencies, resulting in a lack of standardization and reliability in thyroid function tests (54). Secondly, thyroid function is influenced by numerous factors, including nutrition, iodine intake, environmental and geographical factors, genetics, medication use, and various endogenous or exogenous factors (55, 56). Thirdly, thyroid hormone levels can fluctuate throughout the day, and the timing of blood draws may also affect the results (57). Specifically, previous studies show that gender is linked to both TSH and FT4 levels, while weight negatively correlates with FT4 concentration. Ethnicity also affects TSH levels, since gender, weight, and ethnicity may be influenced by genetic factors. Additionally, the timing of venipuncture has a U-shaped relationship with TSH, and the season affects FT4 levels, which are highest in autumn. Higher maternal education is associated with lower FT4 concentrations (58). Thus, standardization in the evaluation of thyroid function and the inclusion of a comprehensive set of thyroid-related parameters could enhance the comparability of findings across different investigations.

Our study also has some disadvantages. Firstly, Thyroid function is influenced by many factors, such as iodine intake, nutrition, medication use, genetics, and socioeconomic status; however, this study lacks comprehensive documentation of these variables. Secondly, the limited patient medical histories complicate thyroid functional status assessment, and the lack of pre-illness thyroid hormone data precludes the establishment of causality. Additionally, we did not have the opportunity to conduct follow-up assessments for children with neurological disorders, limiting our ability to observe potential changes in thyroid function over time.

Our study offers some advantages. Combining the meta-analysis with the case-control study, we present the background of this research in the form of data. It is the first to explore the characteristics of thyroid function tests across the spectrum of neurological disorders with a substantial sample size. Additionally, our study conducted a thorough assessment of various THs, including FT3, FT4, TSH, TT3, TT4, A-TPO, and TG-Ab, enhancing the depth and comprehensiveness of the research. Moreover, the utilization of false discovery rate (FDR) multiple correction serves to reduce false positives in statistical outcomes, while we also considered potential confounding factors such as age and gender.

## 5 Conclusion

Our research demonstrates that, in comparison to controls, children with neurological disorders exhibited a significant decrease in FT4 levels, while TSH levels remained unchanged. This finding provides a reference for potential serological markers of neurological disorders in children. Pediatricians should remain vigilant for hypothyroidism in children with these disorders to enable early detection and intervention, ultimately leading to improved prognoses. Replication in future studies with the assessment of thyroid hormones is needed to determine whether thyroid function should be included as a routine screening in these children.

## Data Availability

The original contributions presented in the study are included in the article/**Supplementary Material**. Further inquiries can be directed to the corresponding author/s.
