# Supplementary material for: Evaluation of thyroid function tests among children with neurological disorders
Source: Front Endocrinol (Lausanne). 2024 Dec 9;15:1498788. doi: 10.3389/fendo.2024.1498788 (PMC11663650; doi:10.3389/fendo.2024.1498788)
Supplement: Supplementary file 1 [file Table1.docx]

**Supplementary Materials**

**Supplementary Tables**

**Table S1** Reference ranges for thyroid function tests in children of different ages

|  | 0-6days | 7days-3months | 4-12months | 1-6years | 7-11years | 12-20years |
| --- | --- | --- | --- | --- | --- | --- |
| FT3 | 2.65-9.68 | 3.00-9.28 | 3.30-8.95 | 3.69-8.46 | 3.88-8.02 | 3.93-7.70 |
| FT4 | 11.00-32 | 11.50-28.30 | 11.50-28.30 | 12.30-22.80 | 12.50-21.50 | 12.60-21.00 |
| TSH | 0.70-15.20 | 0.72-11 | 0.73-8.35 | 0.70-5.97 | 0.60-4.84 | 0.51-4.30 |
| TT3 | 1.12-4.43 | 1.23-4.22 | 1.32-4.07 | 1.42-3.80 | 1.43-3.55 | 1.40-3.34 |
| TT4 | 64.90-239 | 69.60-219 | 73-206 | 76.60-189 | 77.10-178 | 76.10-170 |
| A-TPO | 0-117 | 0-47 | 0-32 | 0-13 | 0-18 | 0-26 |
| TG-Ab | 0-134 | 0-146 | 0-130 | 0-38 | 0-37 | 0-64 |

Note: FT3, free triiodothyronine, (pmol/L); FT4, free thyroxine, (pmol/L); TSH, thyroid-stimulating hormone, (uIU/mL); TT3, total-triiodothyronine, (nmol/L); TT4, total-thyroxine, (nmol/L); A-TPO, thyroid peroxidase antibody, (IU/mL); TG-Ab, anti-thyroglobulin antibodies, (IU/mL).

For newborn babies within six days of birth, the reference intervals were 2.65-9.68 pmol/L for FT3, 11.00-32.00 pmol/L for FT4, 0.70-15.20 uIU/mL for TSH, 1.12-4.43 nmol/L for TT3,64.90-239.00 nmol/L for TT4,0-117.00 IU/mL for A-TPO and 0-134.00 IU/mL for TG-Ab. For infants from seven days of birth to three months, the reference intervals were 3.00-9.28 pmol/L for FT3,11.50-28.30 pmol/L for FT4,0.72-11.00 uIU/mL for TSH,1.23-4.22 nmol/L for TT3,69.60-219.00 nmol/L for TT4,0-47.00 IU/mL for A-TPO and 0-146.00 IU/mL for TG-Ab. For infants from four months to twelve months, the reference intervals were 3.30-8.95 pmol/L for FT3,11.90-25.60 pmol/L for FT4,0.73-8.35 uIU/mL for TSH,1.32-4.07 nmol/L for TT3,73.0-206.00 nmol/L for TT4,0-32.00 IU/mL for A-TPO and 0-130.00 IU/mL for TG-Ab. For babies from one year old to six years old, the reference intervals were 3.69-8.46 pmol/L for FT3,12.30-22.80 pmol/L for FT4,0.70-5.97 uIU/mL for TSH,1.42-3.80 nmol/L for TT3,76.6-189.00 nmol/L for TT4,0-13.00 IU/mL for A-TPO and 0-38.00 IU/mL for TG-Ab. For babies from seven years old to eleven years old, the reference intervals were 3.88-8.02 pmol/L for FT3,12.5-21.5 pmol/L for FT4,0.60-4.84 uIU/mL for TSH,1.43-3.55 nmol/L for TT3,77.10-178.00 nmol/L for TT4,0-18.00 IU/mL for A-TPO and 0-37.00 IU/mL for TG-Ab. For babies from twelve years old to twenty years old, the reference intervals were 3.93-7.70 pmol/L for FT3,12.60-21.00 pmol/L for FT4,0.51-4.30 uIU/mL for TSH,1.40-3.34 nmol/L for TT3,76.10-170.00 nmol/L for TT4,0-26.00 IU/mL for A-TPO and 0-64.00 IU/mL for TG-Ab.

**Table S2** Demographic characteristics of children with neurological disorders compared with healthy controls

|  | Cases  （Mean±SD） | Healthy  （Mean±SD） | | | *P* |
| --- | --- | --- | --- | --- | --- |
| **Tic disorder cases** | | | | | |
|  | n=1067 | n=4801 | | |  |
| Age(years) | 7.17±2.56 | 6.34±3.62 | | | **<0.001** |
| Gender |  |  | | | **<0.001** |
| Male | 840（78.73%） | 2771（57.72%） | | |  |
| Female | 227（21.27%） | 2030（42.28%） | | |  |
| **Attention deficit hyperactivity disorder cases** | | | | | |
|  | n=4864 | n=4801 | | |  |
| Age(years) | 7.24±2.24 | 6.34±3.62 | | | **<0.001** |
| Gender |  |  | | | **<0.001** |
| Male | 3961（81.44%） | 2771（57.72%） | | |  |
| Female | 903（18.56%） | 2030（42.28%） | | |  |
| **Autism spectrum disorder cases** | | | | | |
|  | n=1104 | n=4801 | | |  |
| Age(years) | 2.76±1.35 | 6.34±3.62 | | | **<0.001** |
| Gender |  |  | | | **<0.001** |
| Male | 879（79.62%） | 2771（57.72%） | | |  |
| Female | 225（20.38%） | | 2030（42.28%） |  | |

**Table S3** Laboratory characteristics of males and females including neurological disorders cases and healthy controls

|  | Healthy | Tic disorder cases | | | Attention deficit hyperactivity disorder cases | | | Autism spectrum disorder cases | | |
| --- | --- | --- | --- | --- | --- | --- | --- | --- | --- | --- |
|  | Median (IQR) | Median (IQR) | *P*^*^ | *P*^†^ | Median (IQR) | *P*^*^ | *P*^†^ | Median (IQR) | *P*^*^ | *P*^†^ |
| **Males** | n=2771 | n=840 | | | n=3961 | | | n=879 | | |
| FT3 | 6.710(6.170-7.290) | 6.750(6.230-7.350) | 0.202 | 0.283 | 6.710(6.170-7.280) | 0.930 | 0.963 | 6.850(6.290-7.410) | **<0.001** | **<0.001** |
| FT4 | 18.560(16.980-20.110) | 17.880(16.320-19.480) | **<0.001** | **<0.001** | 17.880(16.480-19.390) | **<0.001** | **<0.001** | 17.830(16.550-19.250) | **<0.001** | **<0.001** |
| TSH | 2.630(1.900-3.580) | 2.680(1.970-3.580) | 0.305 | 0.366 | 2.680(1.930-3.610) | 0.215 | 0.283 | 2.510(1.810-3.420) | **0.012** | **0.027** |
| TT3 | 2.640(2.310-2.980) | 2.420(2.140-2.650) | **<0.001** | **<0.001** | 2.390(2.180-2.680) | **<0.001** | **<0.001** | 2.590(2.310-2.890) | 0.220 | 0.283 |
| TT4 | 113.950(97.978-126.100) | 108.650(93.640-119.430) | **0.029** | 0.053 | 109.800(96.310-120.950) | **0.020** | **0.040** | 111.250(97.750-121.580) | 0.163 | 0.245 |
| A-TPO | 9.355(6.968-12.290) | 10.470(7.790-13.100) | **0.045** | 0.079 | 9.750(6.510-12.310) | 0.944 | 0.963 | 9.780(6.330-12.440) | 0.963 | 0.963 |
| TG-Ab | 12.975(10.450-14.763) | 11.750(7.070-13.590) | **0.001** | **0.003** | 12.400(10.440-15.350) | 0.692 | 0.807 | 7.070(7.070-13.010) | **<0.001** | **<0.001** |
| **Females** | n=2030 | n=227 | | | n=903 | | | n=225 | | |
| FT3 | 6.615(6.020-7.160) | 6.600(6.060-7.110) | 0.955 | 0.955 | 6.590(6.080-7.230) | 0.208 | 0.338 | 6.760(6.180-7.280) | **0.016** | **0.045** |
| FT4 | 18.300(16.698-20.063) | 17.880(16.450-19.370) | **0.011** | **0.036** | 18.090(16.620-19.810) | **0.016** | **0.045** | 17.730(16.320-19.170) | **<0.001** | **<0.001** |
| TSH | 2.490(1.780-3.410) | 2.430(1.870-3.410) | 0.897 | 0.919 | 2.500(1.790-3.470) | 0.802 | 0.864 | 2.190(1.630-3.140) | **0.004** | **0.024** |
| TT3 | 2.530(2.280-2.890) | 2.210(2.010-2.490) | **<0.001** | **<0.001** | 2.430(2.140-2.720) | **0.009** | **0.034** | 2.550(2.260-2.740) | 0.501 | 0.619 |
| TT4 | 111.750(99.143-125.250) | 107.850(95.470-118.250) | 0.251 | 0.387 | 109.100(96.660-121.100) | 0.204 | 0.338 | 109.700(103.300-123.200) | 0.775 | 0.857 |
| A-TPO | 10.195(7.773-13.378) | 11.810(8.980-15.820) | 0.122 | 0.244 | 10.680(7.810-14.290) | 0.258 | 0.387 | 10.100(7.270-14.030) | 0.613 | 0.715 |
| TG-Ab | 13.030(10.340-15.455) | 12.550(7.070-14.610) | 0.190 | 0.338 | 12.720(10.420-16.19) | 0.698 | 0.792 | 10.940(7.070-13.350) | **0.002** | **0.017** |

*P* <0.05 was considered statistically significant. ^*^ Wilcoxon Rank Sum Test. ^†^ Benjamini-Hochberg test

Note: FT3, free triiodothyronine, (pmol/L); FT4, free thyroxine, (pmol/L); TSH, thyroid-stimulating hormone, (uIU/mL); TT3, total-triiodothyronine, (nmol/L); TT4, total-thyroxine, (nmol/L); A-TPO, thyroid peroxidase antibody, (IU/mL); TG-Ab, anti-thyroglobulin antibodies, (IU/mL).

**Table S4** Laboratory characteristics of children including neurological disorders cases and healthy controls

|  | Healthy | Tic disorder cases | | | | | | Attention deficit hyperactivity disorder cases | | | | | Autism spectrum disorder cases | | | | |
| --- | --- | --- | --- | --- | --- | --- | --- | --- | --- | --- | --- | --- | --- | --- | --- | --- | --- |
|  | Median (IQR) | Median (IQR) | *P*^*^ | | *P*^†^ | | | Median (IQR) | | *P*^*^ | | *P*^†^ | Median (IQR) | | *P*^*^ | | *P*^†^ |
| infants | n=700 | n=5 | | | | | | n=7 | | | | | n=103 | | | | |
| FT3 | 6.860(6.320-7.390) | 7.300(6.605-7.905) | | 0.272 | | 0.502 | | 7.290(7.100-8.000) | **0.030** | | 0.145 | | 6.910(6.350-7.550) | 0.541 | | 0.710 | |
| FT4 | 17.570(16.023-19.298) | 17.560(16.240-23.955) | | 0.605 | | 0.762 | | 17.640(16.350-18.910) | 0.978 | | 0.978 | | 17.710(16.780-19.480) | 0.230 | | 0.502 | |
| TSH | 2.640(1.873-3.810) | 2.680(1.635-2.865) | | 0.437 | | 0.656 | | 2.800(2.050-3.650) | 0.733 | | 0.880 | | 2.350(1.750-3.230) | **0.031** | | 0.145 | |
| TT3 | 2.810(2.460-3.120) |  | | 0.149 | | 0.391 | |  | 0.773 | | 0.902 | | 2.570(2.330-2.950) | **0.025** | | 0.145 | |
| TT4 | 118.300(105.300-131.800) |  | | **0.020** | | 0.140 | |  | 0.224 | | 0.502 | | 111.500(97.725-121.000) | **0.044** | | 0.185 | |
| A-TPO | 8.390(6.410-10.980) |  | | 0.282 | | 0.502 | |  | 0.338 | | 0.546 | | 11.030(6.555-13.315) | 0.068 | | 0.260 | |
| TG-Ab | 10.460(7.071-12.490) |  | | 0.922 | | 0.944 | |  | 0.852 | | 0.942 | | 7.071(7.071-12.435) | 0.254 | | 0.502 | |
|  | | | | | | | | | | | | | | | | | |
| toddler period | n=465 | n=57 | | | | | | n=225 | | | | | n=800 | | | | |
| FT3 | 6.770(6.215-7.420) | 6.860(6.220-7.370) | | 0.936 | | 0.980 | 6.700(6.175-7.560) | | | 0.722 | 0.978 | | 6.830(6.233-7.398) | 0.411 | | 0.893 | |
| FT4 | 18.850(17.185-20.415) | 17.720(16.400-19.380) | | **0.008** | | 0.067 | 18.270(16.920-19.930) | | | **0.035** | 0.182 | | 17.655(16.413-19.048) | **<0.001** | | **<0.001** | |
| TSH | 2.590(1.900-3.475) | 2.670(1.920-3.510) | | 0.545 | | 0.914 | 2.740(1.835-3.970) | | | 0.222 | 0.666 | | 2.390(1.710-3.420) | **0.046** | | 0.193 | |
| TT3 | 2.550(2.290-2.880) | 2.635(2.388-2.760) | | 0.870 | | 0.980 | 2.740(2.400-2.905) | | | 0.384 | 0.893 | | 2.590(2.295-2.880) | 0.870 | | 0.980 | |
| TT4 | 111.200(103.400-125.000) | 109.500(96.058-119.275) | | 0.425 | | 0.893 | 118.400(111.900-132.950) | | | **0.024** | 0.144 | | 110.200(98.490-123.000) | 0.340 | | 0.893 | |
| A-TPO | 10.870(7.820-13.700) | 11.230(7.065-13.927) | | 0.913 | | 0.980 | 11.440(7.725-13.435) | | | 0.885 | 0.980 | | 9.490(6.170-12.170) | **0.020** | | 0.140 | |
| TG-Ab | 12.970(10.510-14.910) | 11.750(9.553-16.360) | | 0.691 | | 0.978 | 12.720(10.235-16.370) | | | 0.954 | 0.980 | | 7.071(7.071-13.000) | **<0.001** | | **<0.001** | |
| preschoolers | n=1728 | n=547 | | | | | | n=2616 | | | | | n=193 | | | | |
| FT3 | 6.700(6.123-7.280) | 6.750(6.240-7.340) | | 0.139 | | 0.229 | | 6.710(6.153-7.300) | 0.355 | | 0.497 | | 6.870(6.300-7.330) | **0.018** | | 0.050 | |
| FT4 | 18.870(17.420-20.440) | 18.390(17.030-19.690) | | **<0.001** | | **<0.001** | | 18.170(16.890-19.700) | **<0.001** | | **<0.001** | | 18.390(16.820-19.560) | **<0.001** | | **<0.001** | |
| TSH | 2.660(1.930-3.570) | 2.700(2.000-3.650) | | 0.298 | | 0.432 | | 2.700(1.990-3.630) | 0.190 | | 0.296 | | 2.650(1.925-3.400) | 0.574 | | 0.709 | |
| TT3 | 2.550(2.240-2.890) | 2.380(2.070-2.610) | | **0.003** | | **0.011** | | 2.430(2.220-2.695) | **0.037** | | 0.091 | | 2.570(2.280-2.700) | 0.647 | | 0.753 | |
| TT4 | 117.500(100.600-127.000) | 108.950(95.113-121.525) | | 0.116 | | 0.203 | | 110.500(98.280-123.900) | 0.098 | | 0.194 | | 110.100(97.820-120.100) | 0.106 | | 0.194 | |
| A-TPO | 10.790(7.640-14.860) | 11.140(8.240-13.710) | | 0.992 | | 0.992 | | 9.810(6.550-12.540) | **0.010** | | **0.030** | | 10.750(7.270-13.460) | 0.676 | | 0.753 | |
| TG-Ab | 14.580(12.690-17.580) | 11.795(7.071-13.690) | | **<0.001** | | **<0.001** | | 12.680(10.800-15.920) | **<0.001** | | **<0.001** | | 10.710(7.071-13.750) | **<0.001** | | **<0.001** | |
|  | | | | | | | | | | | | | | | | | |
| school children | n=1535 | n=401 | | | | | | n=1804 | | | | | n=7 | | | | |
| FT3 | 6.560(6.060-7.080) | 6.640(6.155-7.220) | | **0.036** | | 0.086 | | 6.650(6.130-7.210) | **0.003** | | **0.010** | | 5.840(5.170-6.800) | **0.030** | | 0.076 | |
| FT4 | 18.470(16.860-20.070) | 17.400(15.720-19.065) | | **<0.001** | | **<0.001** | | 17.585(16.210-19.208) | **<0.001** | | **<0.001** | | 19.070(16.850-19.640) | 0.892 | | 0.942 | |
| TSH | 2.540(1.830-3.430) | 2.590(1.920-3.505) | | 0.531 | | 0.776 | | 2.580(1.833-3.510) | 0.455 | | 0.692 | | 3.200(2.970-4.380) | 0.112 | | 0.232 | |
| TT3 | 2.490(2.230-2.710) | 2.400(2.045-2.600) | | 0.052 | | 0.116 | | 2.320(2.100-2.550) | **0.002** | | **0.007** | |  |  | |  | |
| TT4 | 104.800(92.140-118.300) | 107.200(93.990-118.550) | | 0.583 | | 0.791 | | 107.100(94.393-117.550) | 0.711 | | 0.821 | |  |  | |  | |
| A-TPO | 14.230(12.830-15.690) | 10.700(8.700-12.965) | | 0.882 | | 0.942 | | 10.100(7.268-12.713) | 0.178 | | 0.338 | |  |  | |  | |
| TG-Ab | 10.370(8.320-12.320) | 11.840(7.071-13.760) | | **<0.001** | | **<0.001** | | 11.700(7.803-14.828) | **<0.001** | | **<0.001** | |  |  | |  | |
|  | | | | | | | | | | | | | | | | | |
| adolescents | n=373 | n=57 | | | | | | n=212 | | | | | n=1 | | | | |
| FT3 | 6.390(5.760-7.045) | 6.590(5.940-7.375) | | 0.051 | | 0.186 | | 6.705(6.185-7.255) | **<0.001** | | **<0.001** | |  |  | |  | |
| FT4 | 17.360(15.675-19.180) | 16.500(14.455-18.090) | | **0.004** | | **0.023** | | 15.970(14.468-17.653) | **<0.001** | | **<0.001** | |  |  | |  | |
| TSH | 2.150(1.620-3.010) | 2.280(1.670-3.115) | | 0.658 | | 0.832 | | 2.290(1.733-3.145) | 0.169 | | 0.418 | |  |  | |  | |
| TT3 | 2.225(1.895-2.485) | 2.345(2.083-2.650) | | 0.240 | | 0.442 | | 2.155(1.973-2.650) | 0.689 | | 0.832 | |  |  | |  | |
| TT4 | 98.280(88.623-110.825) | 110.250(91.570-117.300) | | 0.207 | | 0.426 | | 95.035(72.735-105.725) | 0.229 | | 0.442 | |  |  | |  | |
| A-TPO | 10.195(7.995-14.245) | 10.480(8.110-14.935) | | 0.919 | | 0.952 | | 10.055(5.438-13.020) | 0.314 | | 0.523 | |  |  | |  | |
| TG-Ab | 14.290(13.048-18.588) | 13.120(7.968-15.288) | | 0.191 | | 0.418 | | 11.085(7.071-13.535) | **0.001** | | **0.007** | |  |  | |  | |

*P* <0.05 was considered statistically significant. ^*^ Wilcoxon Rank Sum Test. ^†^ Benjamini-Hochberg test

Note: FT3, free triiodothyronine, (pmol/L); FT4, free thyroxine, (pmol/L); TSH, thyroid-stimulating hormone, (uIU/mL); TT3, total-triiodothyronine, (nmol/L); TT4, total-thyroxine, (nmol/L); A-TPO, thyroid peroxidase antibody, (IU/mL); TG-Ab, anti-thyroglobulin antibodies, (IU/mL).

The infants were under the age of 1 year. Toddler period babies were from 1 to 3 years old. Preschoolers were aged from 3 to 7 years old. School children were from 7 to 11 years old. Adolescents were aged from 11 to 18 years old
